# Supplementary material for: N-type organic thermoelectrics: demonstration of ZT > 0.3
Source: Nat Commun. 2020 Nov 10;11:5694. doi: 10.1038/s41467-020-19537-8 (PMC7655812; doi:10.1038/s41467-020-19537-8)
Supplement: Supplementary file 1 — Supplementary information [file 41467_2020_19537_MOESM1_ESM.docx]

**SUPPLEMENTARY INFORMATION**

**N-type Organic Thermoelectrics: Demonstration of *ZT* > 0.3**

Jian Liu^1,*^, Bas van der Zee^1^, Riccardo Alessandri^1,4^, Selim Sami^1,2^, Jingjin Dong^1^, Mohamad I. Nugraha^3^, Alex J. Barker^5^, Sylvia Rousseva^1,2^, Li Qiu^1,2,†^, Xinkai Qiu^1,2^, Nathalie Klasen^1,2^, Ryan C. Chiechi^1,2^, Derya Baran^3^, Mario Caironi^5^, Thomas D. Anthopoulos^3^, Giuseppe Portale^1^, Remco W.A. Havenith^1,2,6^, Siewert J. Marrink^1,4^, Jan C. Hummelen^1,2^, and L. Jan Anton Koster^1,^*

^1^ Zernike Institute for Advanced Materials, University of Groningen, Nijenborgh 4, 9747 AG Groningen, The Netherlands

^2^ Stratingh Institute for Chemistry, University of Groningen, Nijenborgh 4, 9747 AG Groningen, The Netherlands

^3^ King Abdullah University of Science and Technology (KAUST), Physical Sciences and Engineering Division (PSE), KAUST Solar Center (KSC), Thuwal 23955-6900, Saudi Arabia

^4^ Groningen Biomolecular Sciences and Biotechnology Institute, University of Groningen, Nijenborgh 7, Groningen, NL-9747 AG, The Netherlands

^5^ Center for Nano Science and Technology @PoliMi, Istituto Italiano di Tecnologia, via Pascoli 70/3, 20133 Milano (MI), Italy

^6^ Ghent Quantum Chemistry Group, Department of Inorganic and Physical Chemistry, Ghent University, Krijgslaan 281 (S3), B-9000 Gent, Belgium

^†^ Present address: Yunnan Key Laboratory for Micro/Nano Materials & Technology, National Center for International Research on Photoelectric and Energy Materials, School of Materials and Energy, Yunnan University, Kunming 650091, P. R. China.

*To whom correspondence should be addressed

E-mail: Jian.liu@rug.nl; l.j.a.koster@rug.nl

**Supplementary Methods**

**Supplementary Method 1: GIWAXS measurement**

Grazing incidence wide-angle X-ray scattering (GIWAXS) measurements were performed using a MINA X-ray scattering instrument built on a Cu rotating anode source (λ=1.5413 Å). 2D patterns were collected using a Vantec500 detector (1024x1024 pixel array with pixel size 136x136 microns) located 93 mm away from the sample. The polymer films were placed in reflection geometry at certain incident angles α_i_ with respect to the direct beam using a Huber goniometer. GIWAXS patterns were acquired using incident angles from 0.15°. The direct beam center position on the detector and the sample-to-detector distance was calibrated using the diffraction rings from standard silver behenate and Al_2_O_3_ powders. All the necessary corrections for the GIWAXS geometry were applied to the raw patterns using the GIXGUI Matlab toolbox. The reshaped GIWAXS patterns, taking into account the inaccessible part in reciprocal space (wedge-shaped corrected patterns), are presented as a function of the vertical and parallel scattering vectors q_z_ and q_r_. The scattering vector coordinates for the GIWAXS geometry are given by:

$q=\left\{ \begin{aligned} q_{x}=\frac{2\pi}{\lambda}\left( cos(2\theta_{f})\cos\left( \alpha_{f} \right)-cos(\alpha_{i}) \right) \\ q_{y}=\frac{2\pi}{\lambda}\left( \sin\left( 2\theta_{f} \right)\cos\left( \alpha_{f} \right) \right) \\ q_{z}=\frac{2\pi}{\lambda}\left( \sin\left( \alpha_{i} \right)+sin(\alpha_{f}) \right) \end{aligned} \right.$ (Supplementary Equation 1)

where $2\theta_{f}$ is the scattering angle in the horizontal direction and $\alpha_{f}$ is the exit angle in the vertical direction. The parallel component of the scattering vector is thus calculated as $q_{r}=\sqrt{q_{x}^{2}+q_{y}^{2}}$.

**Supplementary Method 2: Free charge carrier measurement**

The MIS devices have an architecture of ITO/insulator/doped fullerene derivative film/Al. For ion gel solution preparation, 251 mg PVDF-HFP was dissolved in 3.17 mL cyclohexanone and stirred at 70 ^o^C at 1000 rpm overnight. In a sequential process, 91 mg [EMIM][TFSI] was added into the solution and stirred at 55 ^o^C for at least four hours. Before spin-coating, the temperature of ion gel was set to 40 ^o^C one hour. Finally, the ion gel solution was spin-coating on clean ITO substrates to form 150-300 nm insulator layer followed by annealing at 140 ^o^C for 4 hours. 5 wt%-doped PTEG-2 films were prepared by spin-coating with a thickness of around 100 nm on top of insulators and resulting devices were annealed at 120 oC and 150 ^o^C, respectively. 100 nm-thick Al was deposited in vacuum as the top electrode to finish the fabrication of MIS devices. The capacitance-voltage (*C*_p_-*V*) measurement was conducted at a frequency of 10 Hz for ion gel based devices for AC bias. The carrier density (*n*) was extracted by Mott-Schottky analysis:^1^

$n=\frac{2}{e\varepsilon_{0}\varepsilon_{r}\frac{\partial C_{P}^{-2}}{\partial V}}$ (Supplementary Equation 2)

Where *e*, *ε*_0_, and *ε*_r_ are elementary charge, dielectric constant of vacuum and relative dielectric constant of active layer, respectively. *ε*_r_=4.5 was used for both doped layers.

**Supplementary Notes**

**Supplementary Note 1: In-situ dynamic ellipsometry**

**Variable angle Ellipsometric Spectroscopy**. Various fullerene derivative films were prepared on clean silicon substrates with a thin layer of native oxide under the spin-coating conditions with the device fabrication. The prepared fullerene derivative based thin films were placed in a vacuum chamber (<10^-6^ par) for two days in order to remove any trapped solvent. The thin-film samples were placed in an air-protected sample holder with continuous N_2_ flow and the sample holder was amounted on a variable angle ellipsometer (J. A. Wollam Co., inc) for the optical measurements. Firstly, the spectroscopic scan (from 300 nm to 1700 nm) was conducted for each sample at room temperature.

**The model and fits.** The model for fitting the spectroscopic data of all the samples: Si (substrate)/SiO_2_/Chaucy layer. The thickness of SiO_2_ layer was obtained by fitting the spectroscopic data, which is 3.6 nm. The Cauchy function (*n*(λ)=An+Bn/λ^2^+Cn/λ^4^, *k*=0, where *n* is the refractive index, *k* extinction coefficient, An, Bn and Cn are Cauchy parameters) was used as the dispersion function of the fullerene derivative based thin films. The least-squares fitting to ellipsometry data in the region where *k* approaches the zero (from 700 nm to 1700 nm) was performed for determining the original film thickness (*d*) and the Cauchy parameters at 25 ^o^C, which are displayed in Supplementary Table 1. The morphology study in Supplementary Fig. 3 indicates that there are aggregations on the surface of the doped PTEG-2 films. In order to take into account this point, We also used a bilayer model for the doped PTEG-2 film and the bilayer includes a Cauchy layer and an atop composite layer consisting of the Cauchy material and void with a certain percentage (*f*). The thickness (*d*2) of the composite layer was determined by the AFM result and set to 50 nm. The fitting was only slightly improved with MSE of 0.4 relative to MSE=1.29 by the one layer model. By fitting the experimental data, the thickness (*d*1) of the Cauchy layer and the percentage of the void were determined to be 89.7 nm and 96.4%, respectively. Therefore, the volume-translated thickness (*d*) of organic material could be expressed by: *d*=*d*2+*d*1• (1-*f*) and we obtain *d*=91.5 nm, which is very close to *d*=91.6 fitted by the one layer model. Therefore, we think the one layer model provides a good approximation for the volume-translated thickness for the doped PTEG-2.

**Dynamic spectroscopy**. For monitoring the phase behavior of the film samples, temperature ramp scan with a rate of 2.5 ^o^C/minute was carried out from 25 ^o^C to 200 ^o^C at a wavelength of 800 nm and an incident angle of 70^o^. Supplementary Fig. 1 displays plots of the ellipsometry parameter ψ as a function of the temperature *T* for pristine and doped fullerene derivatives films. Previous work reached an important empirical law that for relatively small changes in morphology and thickness, such as those normally caused by thermal annealing, the ellipsometry parameters typically vary approximately linearly with film thickness.^2^ Based on this empirical law, the fitted thickness *d* and ψ(T), we could derive the evolution of *d* with *T* for each thin film samples, which are displayed in Fig. 2a. For a given fullerene derivative-based film which does not undergo decomposition at this temperature range, the thickness inversely scales with the film density. As such, the slope of *d* (T) at a certain T is proportional to the linear thermal expansion coefficient, which reflects the phase behavior of the organic film.

**Supplementary Table 1** **The model fit.** The summary of fitting parameters.

| **Model: Si/SiO_2_/Cauchy layer; fit wavelength range: 700 nm - 1700 nm** | | | | | | |
| --- | --- | --- | --- | --- | --- | --- |
| Samples | An | Bn | Cn | *d* (nm) | | **MSE** |
| Pristine PTEG-2 | 1.796 | 0.008860 | 0.0135 | 89.8 | | **2.68** |
| Pristine F2A | 1.760 | 0.00736 | 0.0115 | 92.4 | | **11.0** |
| Pristine PTEG-1 | 1.872 | -0.00708 | 0.02734 | 93.8 | | **0.80** |
| Pristine PPEG-1 | 1.830 | -0.0078 | 0.0266 | 92.3 | | **0.61** |
| Doped PTEG-2 | 1.836 | -0.0344 | 0.0241 | **91.6** | | **1.29** |
| **Model: Si/SiO_2_/Cauchy layer(*d*1)/(1-*f*) Cauchy+*f* Void (set *d*2=50 nm)**  **Fitting wavelength range: 700 nm - 1700 nm** | | | | | | |
|  | An | Bn | Cn | *d*1 (nm) | *f* | **MSE** |
| Doped PTEG-2 | 1.884 | -0.0592 | 0.0253 | 89.7 | 0.964 | **0.40** |
|  |  |  |  | *d*=*d*1+*d*2×(1-*f*)= **91.5** | |  |



 **Supplementary Fig. 1 The model fits.** Experimental ellipsometry data and model fits for **a** pristine PTEG-2, **b** pristine F2A, **c** pristine PTEG-1, **d** pristine PPEG-1, **e** as-cast doped PTEG-2 thin film at 25 ^o^C; **f** the plots of ellipsometry parameter ψ as a function of temperature T for various pristine and doped fullerene derivative films.

**Supplementary Note 2: TGA for fullerene derivatives**

The fullerene derivatives and n-DMBI powder samples were dried in a vacuum oven overnight. 1-3 mg sample was taken for thermogravimetric analysis (TGA) testing. During the measurement, temperature ramped at 10^o^C/min to 700^o^C. The decomposition temperature is taken at 95% weight. From the TGA plots in Supplementary Fig. 2, the decomposition temperatures of PTEG-1, PPEG-1, PTEG-2, and F2A are 300^o^C, 296^o^C, 347^o^C and 347^o^C, respectively. n-DMBI first undergoes slight weight gain above 90 ^o^C, which is due to the oxidation, and then keeps the weight until 200 ^o^C, and degrades afterwards.

**
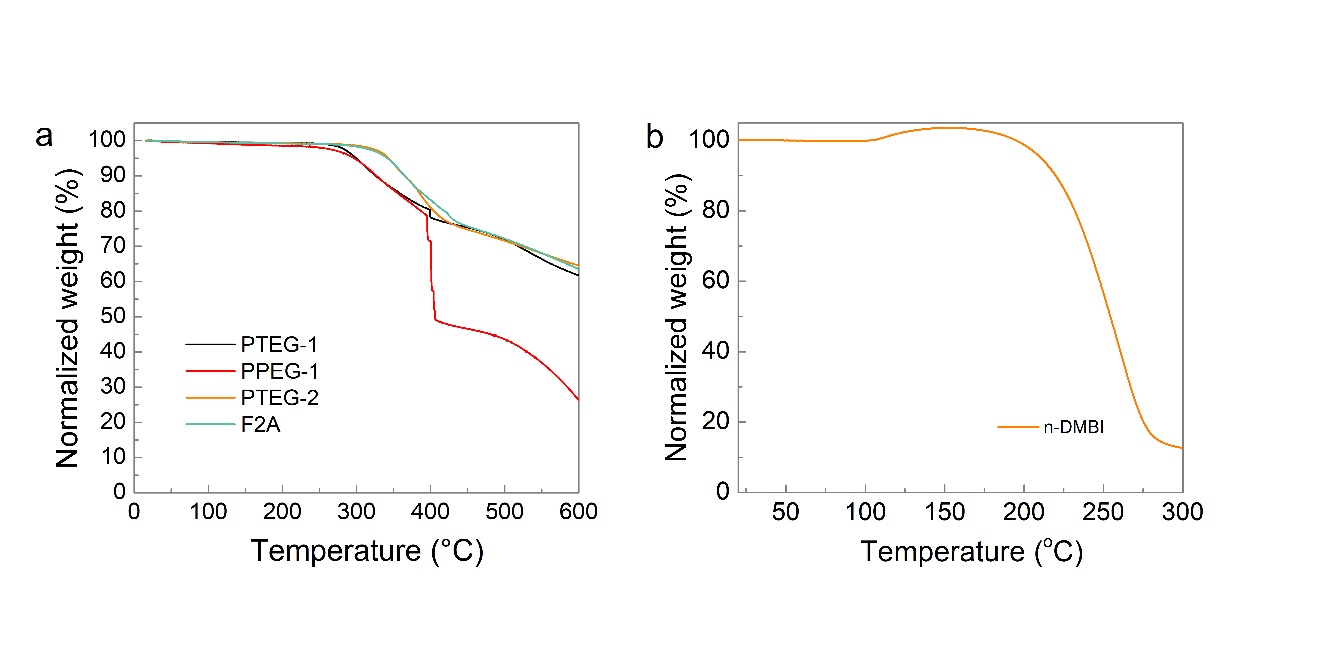
**

**Supplementary Fig. 2 Thermal stability of powder sample.** Thermogravimetric analysis (TGA) plots of (**a**) pristine PTEG-1, PPEG-1, PTEG-2 and F2A, and (**b**) n-DMBI.


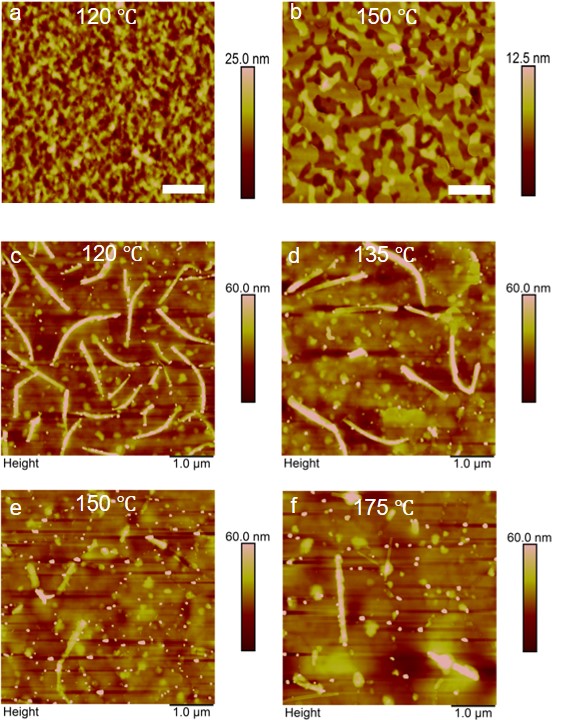


**Supplementary Fig. 3** **Surface Morphology.** AFM images for pristine PTEG-2 film upon annealing at 120 ^o^C (**a)** and at 150 ^o^C (**b**); **c-e** the evolution of surface morphology of 8 wt%-doped PTEG-2 film with the annealing temperature T (120 ^o^C, 135 ^o^C, 150 ^o^C and 175 ^o^C).

**
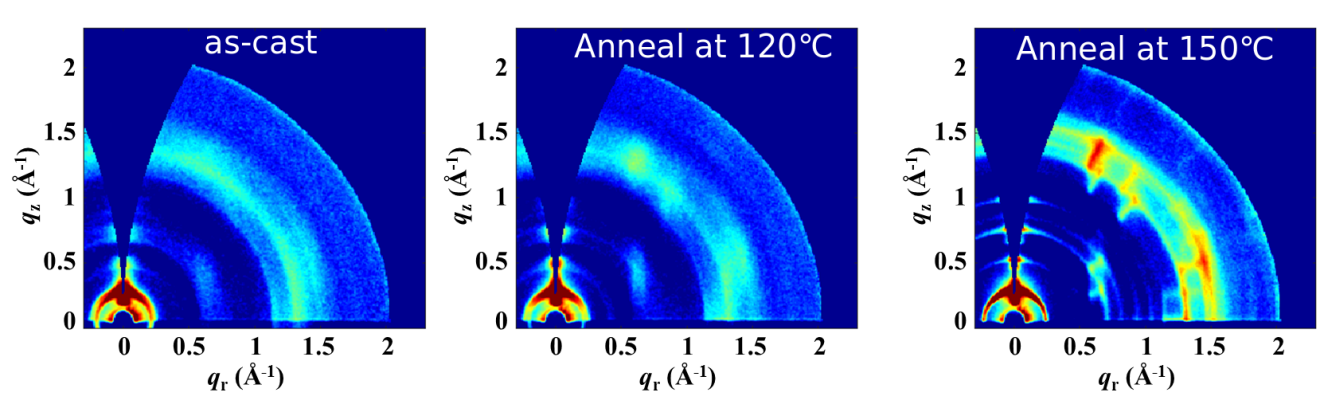
**

**Supplementary Fig. 4 Molecular packing.** Two dimensional GIWAXS patterns of doped PTEG-2 films at various annealing conditions (non-annealing, and annealed at 120 ^o^C and 150 ^o^C, respectively).

**Supplementary Note 3: Molecular Dynamics simulations**

**Force Field.** The employed force field is the one developed and thoroughly described in a previous study.^3^ Briefly, the force field uses Lennard-Jones (LJ) parameters from the GROMOS 54A6 parameter set,^4^ based on previous work,^5^ which is improved by the derivation of bonded parameters (bond, angle, and dihedrals parameters) from quantum chemical calculations, following the Q-Force procedure.^6^

**Protocol.** The starting configuration of the molecular dynamics (MD) simulations include 2 molecules in an orthorhombic unit cell with dimensions of about 1 x 1 x 5 nm^3^. The following 6 MD simulations were then carried out in series on such starting configuration: 1) 698.15 K, 200 bar, harmonic constraint of 500 kJ mol^-1^ nm^-2^; 2) 298.15 K, 200 bar, harmonic constraint; 3) 298.15 K, 1 bar, harmonic constraint; 4) 298.15 K, 1 bar; 5) 598.15 K, 1 bar; 6) 298.15 K, 1 bar. This leads to a total of 6 ns of MD simulations (1 ns per step). The harmonic constraint, when present, keeps the two C_60_s at a distance of 1 nm. In this first part of the protocol, due to the limited size of the unit cell, shorter cutoffs must be used for LJ and Coulomb interactions (0.45 nm). Then, the unit cells obtained were replicated 5 times along the a and b directions and 3 times along the c direction, leading to simulation boxes of about 5 x 5 x 8 nm^3^. Three further MD simulations were then carried out: 7) 298.15 K, 100 bar 1 ns; 8) 298.15 K, 1 bar, 1 ns; 9) 298.15 K, 1 bar, 5 ns. Including the 6 ns of steps 1)-6), this leads to a total of 13 ns of MD simulation. For steps 7)-9), the default cutoff of 1.4 nm for LJ interactions and the Particle Mesh Ewald (PME)^7^ method for electrostatic interactions were used. Weak coupling schemes^4^ were used in steps 1) to 8) to maintain pressure, in an anisotropic way and with a compressibility of 5×10^-6^ bar, and temperature at the different stages of the MD protocol (see above): coupling parameters were of 1 ps and 0.5 ps in the temperature and pressure cases, respectively. The nose-Hoover thermostat^8,9^ and the Parrinello-Rahman barostat^10^ were used to maintain temperature (coupling parameter of 1 ps) and pressure (coupling parameters of 5 ps, anisotropic), respectively, in the step 9). 240 independent MD simulations are run following this protocol. The outcome of the simulations, the *a*, *b*, and *c* axis (which allow to compute the density) were extracted from the last 4 ns of steps 9) and are plotted as histograms in Supplementary Fig. 5.

**Supplementary Fig. 5 Molecular dynamic simulation.** Distribution for the *a*, *b*, and *c* axis (nm) obtained from 240 MD simulations of the unit cell of PTEG-2 (simulation box size of about 5 x 5 x 8 nm^3^). The density (g/cm^3^) is also plotted.

**Simulated Scattering.** The *z* components of the atom coordinates obtained from the MD simulations were binned into a one-dimensional histogram. The unit cell (containing 2 molecules) was replicated 25 times along the *z* coordinate (*c* axis). 1024 data points in real space were used, evenly separated along a 1024 Å length. This occurrence histogram was then convolved with a Lorentzian function with full width at half maximum of 0.2 Å, to smear out the point scatters obtained from the atom coordinates, in this way accounting for the missing electron density. The convoluted histogram was then Fourier transformed by using a Fast Fourier transform algorithm, giving the scattering wave $\psi_{s}$ in the reciprocal space (q)

$\psi_{s}\left( q \right)=\sum_{j=1}^{N} f_{j}e^{-i q R_{j}}$ (Supplementary Equation 3)

where f_j_ is the form factor of atom j, and R_j_ is its coordinate in Z. Hydrogen atoms were skipped during this process and the form factor f_j_ was considered equivalent for the remainder of the atoms. The scattering intensity (I) is then obtained by multiplying the scattering wave with its complex conjugate:

${I\left( q \right)=\psi}_{s}\left( q \right) x \psi_{s}^{*}\left( q \right)$ (Supplementary Equation 4)

These scattering intensities were obtained for the 240 unit cells obtained from the MD simulations and averaged. The final simulated scattering linecut along *q_z_* is obtained by convolving this average with a Gaussian function with standard deviation of 0.02 Å, which adds a minimal amount of heterogeneity, hence slightly smoothening the resulting average linecut, in line with the standard deviation of the different unit cells.

The same procedure was repeated but now using the *y* component of the atom coordinates to obtain the linecut along *q_y_*.

**

**

**Supplementary Fig. 6 Thermoelectric characterization.** (**a**, **b**) The J-V curves and (**c**, **d**) the plots of thermal voltage versus temperature difference for doped PTEG-2 films at different doping concentrations upon annealing at 120 ^o^C and 150 ^o^C, respectively.





**Supplementary Fig. 7 Free charge and polaron generation. a** The *C*_p_-*V* plots of MIS devices based on the 5 wt%-doped PTEG-2 films upon annealing at 120 ^o^C and 150 ^o^C; **b** Mott-Schottky plots and corresponding fits for MIS devices based on the doped PTEG-2 films upon annealing at 120 ^o^C and 150 ^o^C (the error bars were made considering the dielectric constant deviation and fitting error); **c** EPR spectra of pristine and doped PTEG-2 films annealed at different temperatures.

**Supplementary Note 4: In-plane thermal conductivity measured by 3-omega** (3ω) **method**

Thermal conductivity measurement was performed by Linseis thin film analyzer (TFA) setup employing 3-omega (3ω) method. The PTEG-2-based films were prepared by drop-casting or spin-coating the solution on a thermal conductivity measurement chip integrated with two suspended membranes as shown in Supplementary Fig. 8 in a N_2_-filled glovebox. The suspended membranes were transferred into the Linseis setup and kept in vacuum. Before the in-plane thermal conductivity measurement, the samples were thermally annealed at 150 ^o^C to activate the doping process and initiate the phase transition. The thickness of the deposited PTEG-2 based films was monitored by using DEKTAK profilometer. The pristine and doped PTEG-2 films have thickness of 3.71±0.35 µm and 6.71±0.23 µm, respectively.


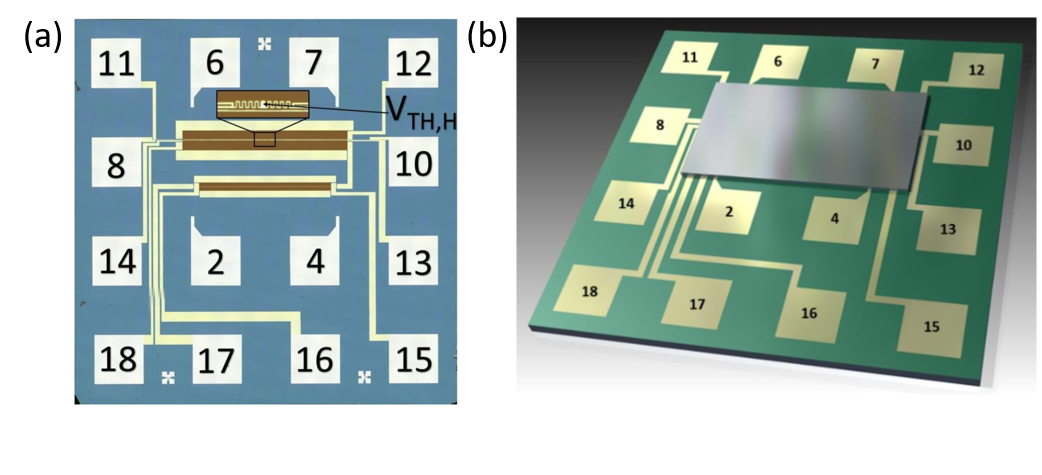


**Supplementary Fig. 8** **Chip architecture.** Chip for thermal conductivity measurement integrated with two suspended membranes.

In the 3ω method, an AC current (*I=I_0_ cos(ωt)*) is applied to the heating stripes, generating an increase in temperature of the membrane (*∆T=∆T_0_ cos (2ωt + φ)*) with phase shift of *φ* and oscillation in the resistance of the stripe *R = R_0_ (1 + β∆T)* at the angular frequency of *2ω. β* is the temperature coefficient of the heating stripes. The voltage drop across the heater (*V_3ω_*) was measured which gives the amplitude of a modulated signal. This amplitude of the modulated signal can be analyzed which has a small component at the third harmonic *3ω*. The general formula for amplitude of the voltage drop is given by,

$$\left| V_{3\omega} \right|=\frac{\beta R_{0}^{2}I_{0}^{3}}{4K_{P}\sqrt{1+\omega^{2}\left( 4\tau^{2}+\frac{w^{4}}{24D^{2}}+\frac{\tau w^{2}}{3D} \right)}}$$

Supplementary Equation 5

Supplementary Equation 6

$$K_{P}=\frac{2\lambda tl}{w}$$

with *R_0_*, *w*, *τ*, and *D* are unloaded resistance of the heater, width of the membrane, thermal relaxation time, and thermal diffusivity of the sample and membrane, respectively. The thermal diffusivity *D* is related to the mass density *ρ_m_*, specific heat capacity *c*, and thermal conductivity *λ* i.e. (*D=λ/ρ_m_c*). The thermal relaxation time *τ* is given by *τ=C‘/K_P_*, with *C‘* is the total specific heat of the membrane. The product *λt* and *C‘* can be extracted by fitting the measured *3ω* voltage versus the frequency using Supplementary Equation 5. At quasi-steady state conditions (at low frequencies), the amplitude of the *3ω* oscillation is given by,

Supplementary Equation 7

$$\left| V_{3\omega} \right|=\frac{\beta R_{0}^{2}I_{0}^{3}}{4K_{P}}$$

The thermal conductivity of the sample *λ_s_* with thickness of *t_s_* can be calculated by subtracting the total measured thermal conductivity and the values of the empty measurement chip (*λ_m_*, *t_m_*),

Supplementary Equation 8

$$\lambda_{s}=\frac{\lambda t-\lambda_{m}t_{m}}{t_{s}}$$

All of the above processes are analyzed in a built-in software given by Linseis Thin Film Analysis (TFA) setup. The details of 3ω thermal conductivity measurement method can be found in these references.^11,12^

**Supplementary Note 5: Thermal conductivity measured by an optical pump-probe technique**

The thermal conductivity of doped PTEG-2 film in the out-of-plane direction was measured by an optical pump-probe spectroscopic technique previously reported^13^, as schemed in Supplementary Fig. 9a, based on the method developed by Capinski and co-workers.^14^ The measurement can be considered a simplified version of time-domain thermoreflectance, one which can be easily performed using typical pump−probe spectroscopy setups, renouncing phase-sensitivity.^15^ To prepare the thin-film sample, doped PTEG-2 films of different thickness were spin-coated on clean silicon substrates following by thermal annealing at 150 ^o^C, and 80-90 nm Al is deposited on top of organic films as back reflective electrode. To avoid degradation due to air exposure, samples were transported under vacuum between spin-coating and Al evaporation, and then kept in inert N_2_ atmosphere (in a small chamber designed for spectroscopy) while transported to the spectroscopy facility. During optical pump-probe measurements, the samples were actively pumped to lower than 10^-5^ torr. In this experiment, a pulse from a Q-switched Nd:YVO4 laser (532 nm, 700 ps FWHM pulse width, 350 μm FWHM spot size) creates a transient increase in the temperature of the aluminum, which then cools by transferring heat through the organic thin-film sample into the silicon substrate (as demonstrated in Supplementary Fig. 9b). In Supplementary Fig. 9c, the variation of reflectivity, △R, of the aluminum deposited on top of the films is shown with respect to time for three samples of doped PTEG-2, characterized by a different thickness. The experimental data (open circles) are fitted (solid lines) considering 1D heat diffusion through the bulk of the three layers (aluminum, doped PTEG-2, and silicon substrate) and through each interface, characterized by an interfacial thermal conductance, with the same parameters of interfacial thermal resistance and material properties of each active layer thickness studied.


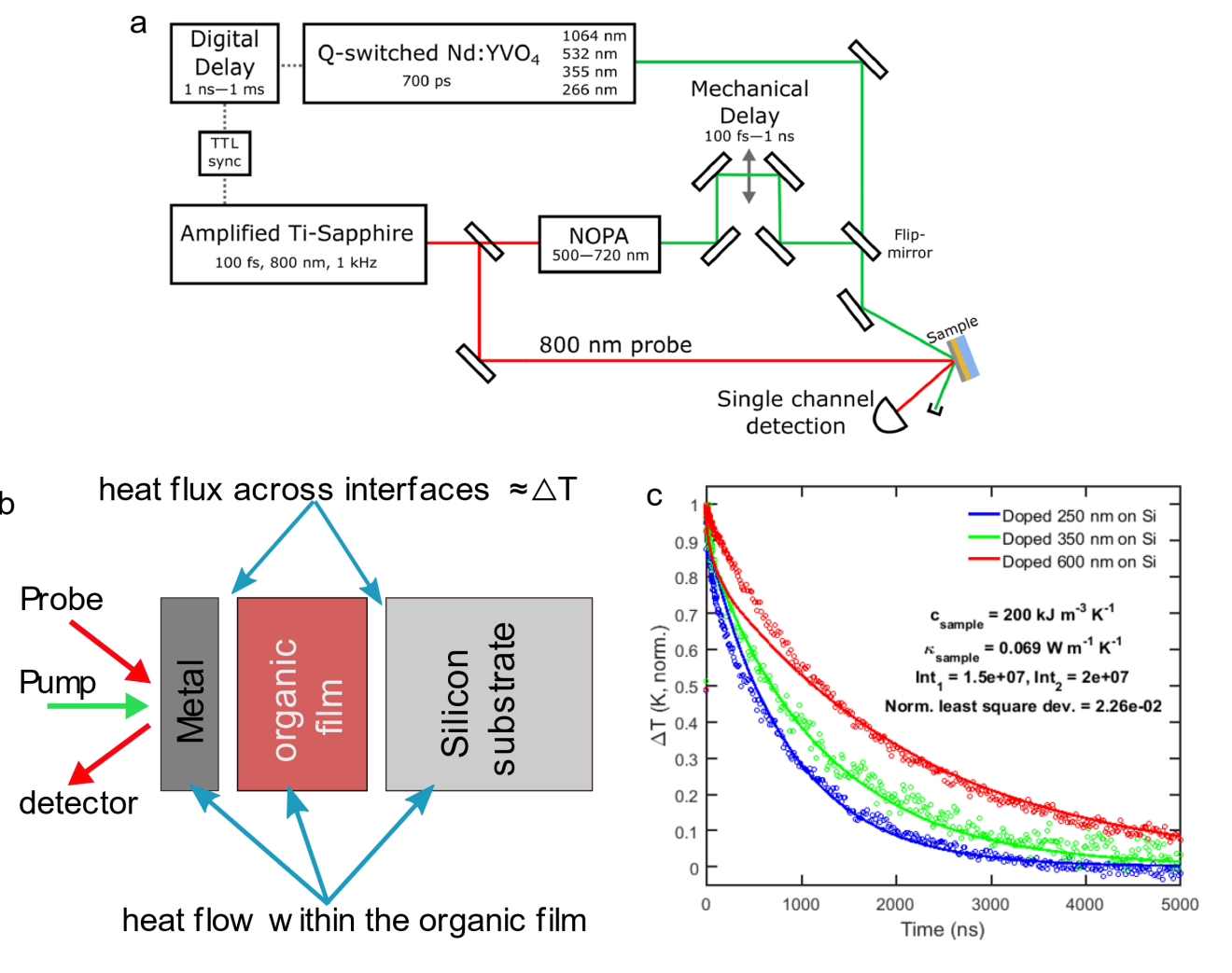


**Supplementary Fig. 9 The out-of-plane thermal conductivity.** **a** The schematic of optical pump-probe spectroscopic setup; **b** Scheme of measurement of the vertical thermal conductivity by optical pump-probe method. If the characteristic dimension of the laser spot is much larger than the thickness of the film, the heat transport parallel to the aluminum film surface can be neglected, and the problem can be treated as one-dimensional (1D); **c** Normalized reflectivity variation with time of the aluminum film for the doped PTEG-2 thin films of varying thickness. The thermal conductivity in the out-of-plane direction is extracted from the fitting of a series of films with different thickness.

**Supplementary Note 6: Measurement of temperature dependence of Conductivity and Seebeck coefficient**

For the electrical conductivity measurement at elevated temperatures, the device of the same geometry as described in Supplementary method for four-point probes measurement was fabricated and placed on a stage with temperature control. The J-V curves of doped PTEG-2 film (upon annealing at 150 ^o^C) at elevated temperatures (from 303 K to 443 K) are displayed in Supplementary Fig. 10a and the electrical conductivities were derived by performing linear fitting.

The Seebeck coefficient of doped organic semiconductor films at elevated temperature was measured on a stage with temperature control. As shown in the Scheme (Supplementary Fig. 10b), the two pairs of Au line electrodes (width: 1 mm and length: 7 mm; width: 1 mm and length: 4 mm) were deposited on a glass substrate with a distance of 7 mm. The thin-film sample was spin-coated on one of the Au line electrodes (width: 1 mm and length: 7 mm) (other area was covered by scotch tape before coating). The standard Constantan wire (127 μm from Omega) was attached on the other of the Au line electrodes (width: 1 mm and length: 4 mm) with silver paste (ELECTRODAG 1415). The temperature difference across the sample was posted by a thin film heater (KFR-5-120-C1-16, KYOWA), which was attached on the side of a glass substrate with a connection part of a small copper block for uniform heat transfer. The heater was controlled by Keithley 2635. The generated thermal voltages from Constantan wire (V_th_ of the reference, V*_ref_*) and thin-film sample (V_th_ of sample, V*_sample_*) were probed by four probes at the same time and corresponding data were recorded by Keithley 2000 with a scanning card. Linearly increased power was input into the thin-film heater to generate the temperature difference and a home-made filter (cut-off frequency=1 HZ) was used for reducing the noise. The temperature of the sample was controlled by the stage with a separate temperature controlling system. The whole system was controlled by Labview software. The Seebeck coefficient (*S*) of sample was obtained by the formula:

$S=\frac{V_{sample}}{V_{ref}}S_{Const}+\left( \frac{V_{sample}}{V_{ref}}-1 \right)S_{Au}$ Supplementary Equation 9

Where *S*_const_ and *S*_Au_ are the Seebeck coefficients of Constantan wire and Au layer. At room temperature, *S*_const_=-39 μV/K and *S*_Au_=1.49 μV/K, respectively. Note the excessive area of thin film was scratched for eliminating geometric artifacts.^14^ The value of V*_sample_*/V*_ref_* is derived by linear fitting the curve of V*_sample_* versus V*_ref_* as demonstrated in Supplementary Figs. 10c and 10d. The temperature dependence of the Seebeck coefficient of Constantan wire was derived as following (the valid temperature range:80 K<T<600 K):^15^

$S_{Con}\left( T \right)=S_{Cu}\left( T \right)-S_{{Cu}/{Con}}\left( T \right)$ Supplementary Equation 10

$S_{{Cu}/{Con}}\left( T \right)=4.37184+0.1676T-1.84371\times{10}^{-4}T^{2}+1.2244\times{10}^{-7}T^{3}-4.47618\times{10}^{-11}T^{4}$ Supplementary Equation 11

$S_{Cu}\left( T \right)=0.041\times T\left[ \exp\left( -\frac{T}{93} \right)+0.123-\frac{0.442}{1+\left( \frac{T}{172.4} \right)^{3}} \right]+0.804,$ Supplementary Equation 12

**
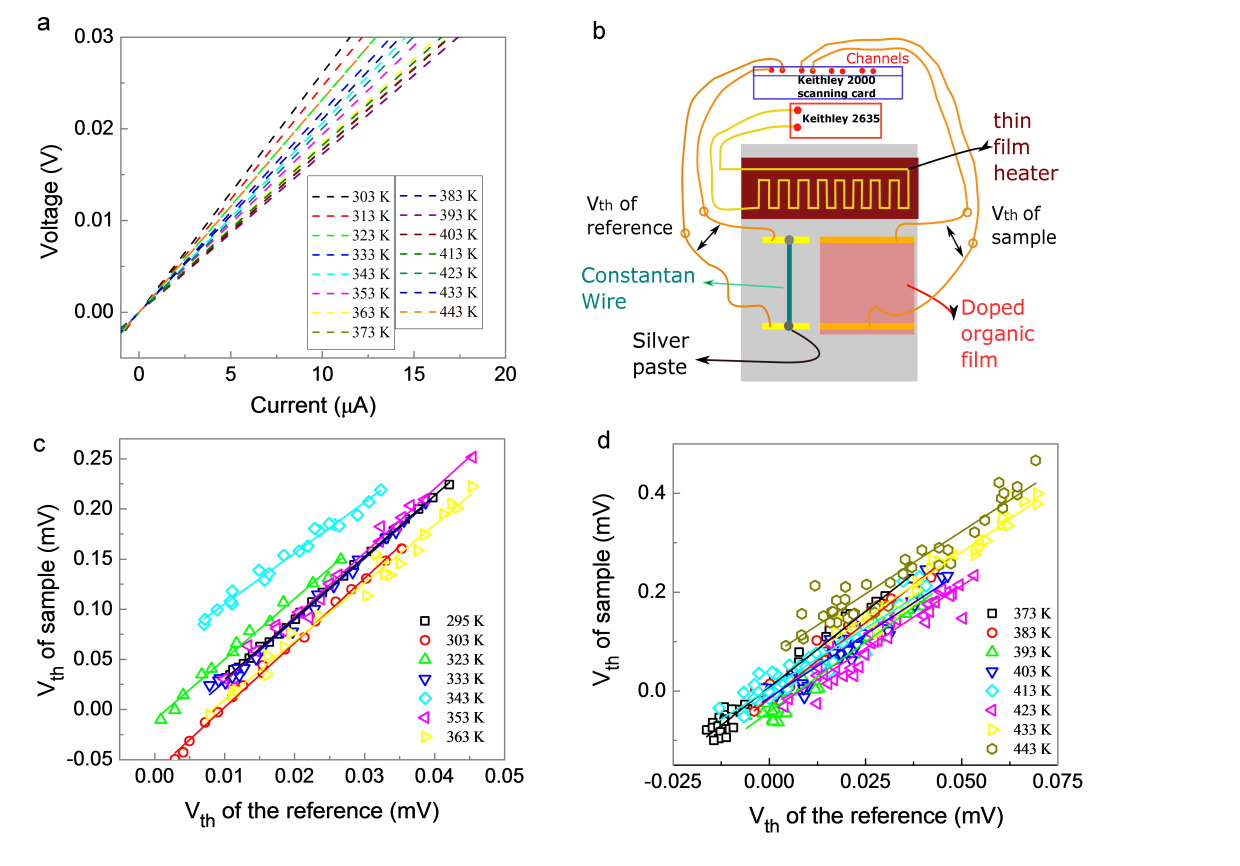
**

**Supplementary Fig. 10 Temperature dependent measurement. a** the J-V curves of doped PTEG-2 film (annealed at 150 ^o^C) at elevated temperatures (from 303 K to 443 K); **b** the scheme of the device architecture and electric connection for the Seebeck coefficient measurement under different *T*; **c** and **d** the plots of thermal voltage (*V*_th_) of sample versus the *V*_th_ of the reference (Constantan wire) measured at different temperatures. The doping concentration for the active layer was kept at 5 wt%.





**Supplementary Fig. 11 Temperature dependent conductivity.**  The Arrhenius plot of the temperature dependent electrical conductivity of the doped PTEG-2 upon annealing at 150 ^o^C. The extracted activation energy *E*_a_=51 meV. (Fitting quality: Adj. R^2^=0.993) **Supplementary References:**

1. Pingel, P., Schwarzl, R. & Neher, D. Effect of molecular p-doping on hole density and mobility in poly(3-hexylthiophene). *Appl. Phys. Lett.* **100**, 143303 (2012).

2. Campoy-Quiles, M., Alonso, M. I., Bradley, D. D. C. & Richter, L. J. Advanced Ellipsometric Characterization of Conjugated Polymer Films. *Adv. Funct. Mater.* **24**, 2116–2134 (2014).

3. Sami, S., Alessandri, R., Broer, R., and Havenith, R. W. A. How ethylene glycol chains enhance the dielectric constant of organic semiconductors: molecular origin and frequency dependence. *ACS Appl. Mater. Interfaces* **12**, 17783-17789 (2020).

4. Berendsen, H. J. C., Postma, J. P. M., van Gunsteren, W. F., DiNola, A. & Haak, J. R. Molecular dynamics with coupling to an external bath. *J. Chem. Phys.* **81**, 3684–3690 (1984).

5. Qiu, L. *et al.* Enhancing doping efficiency by improving host-dopant miscibility for fullerene-based n-type thermoelectrics. *J. Mater. Chem. A* **5**, 21234–21241 (2017).

6. Sami, S., Broer, R., Havenith, R. W. A. Q-Force: QM-Derived polarizable force field parameters. https://github.com/selimsami/qforce. (2019).

7. Russ, B., Glaudell, A., Urban, J. J., Chabinyc, M. L. & Segalman, R. A. Organic thermoelectric materials for energy harvesting and temperature control. *Nat. Rev. Mater.* **1**, 16050 (2016).

8. Nosé, S. A molecular dynamics method for simulations in the canonical ensemble. *Mol. Phys.* **52**, 255–268 (1984).

9. Hoover, W. G. Canonical dynamics: Equilibrium phase-space distributions. *Phys. Rev. A* **31**, 1695–1697 (1985).

10. Otten, M. & Gray, S. K. Recovering noise-free quantum observables. *Phys. Rev. A* **99**, 012338 (2019).

11. Linseis, V., Völklein, F., Reith, H., Woias, P. & Nielsch, K. Analytical Investigation of the Limits for the In-Plane Thermal Conductivity Measurement Using a Suspended Membrane Setup. *J. Electron. Mater.* **47**, 3203–3209 (2018).

12. Linseis, V., Völklein, F., Reith, H., Nielsch, K. & Woias, P. Advanced platform for the in-plane ZT measurement of thin films. *Rev. Sci. Instrum.* **89**, (2018).

13. Beretta, D. *et al.* Thermoelectric Properties of Highly Conductive Poly(3,4-ethylenedioxythiophene) Polystyrene Sulfonate Printed Thin Films. *ACS Appl. Mater. Interfaces* **9**, 18151–18160 (2017).

14. Reenen, S. van & Kemerink, M. Correcting for contact geometry in Seebeck coefficient measurements of thin film devices. *Org. Electron.* **15**, 2250–2255 (2014).

15. Guan, A. *et al.* An experimental apparatus for simultaneously measuring Seebeck coefficient and electrical resistivity from 100 K to 600 K. *Rev. Sci. Instrum.* **84**, 043903 (2013).
